# Age discrimination perceived by hospitalized older adult patients in Iran: A qualitative study

**DOI:** 10.34172/hpp.2022.07

**Published:** 2022-05-29

**Authors:** Sakineh Gholamzadeh, Maryam Shaygan, Zeinab Naderi, Fahimeh Alsadat Hosseini

**Affiliations:** ^1^Community Based Psychiatric Care Research Center, School of Nursing and Midwifery, Shiraz University of Medical Sciences, Shiraz, Iran; ^2^Department of Nursing, Sirjan School of Medical Sciences, Sirjan, Iran

**Keywords:** Ageism, Aging, Healthcare disparities, Perceived discrimination

## Abstract

**Background:** The quality of care that older adult patients receive during hospitalization is directly associated with the perception, knowledge, and skills of the healthcare team. This qualitative study was conducted to explore the concept of age discrimination perceived by hospitalized older adult patients.

**Methods:** The present exploratory qualitative study was conducted using conventional content analysis. The purposive sampling method was used to recruit participants and the sampling continued until data saturation. A total of 21 individuals comprising of 12 hospitalized older adult patients, 5 family caregivers, 3 nurses, and a physician were enrolled in the study. Data were collected through 21 face-to-face, semi-structured, in-depth interviews. Data were analyzed using the qualitative content analysis method as described by Elo and Kyngäs.

**Results:** The analysis of the interview data resulted in 4 main categories, namely injustice perceptions, interactional injustice, procedural injustice, and organizational injustice.

**Conclusion:** The findings of the study indicated that older adult patients perceive the occurrence of age discrimination by healthcare teams and inequalities in the provided care in hospitals. It is therefore important to address ageism and subsequent inequalities through short- and long-term policies and plans, as well as standardization and transformation of the present condition of hospitals to become an age-friendly environment.

## Introduction

The population of older people is rapidly growing worldwide. It is estimated that the population over the age of 60 will increase from 900 million in 2015 to 2 billion in 2050.^1^ While population aging started in high-income countries, by 2050, 80% of the people aged 60 years and older will be living in low- and middle-income countries.^2^ Because of this demographic shift, there is an increased need for the provision of dedicated care for older adults and adaptation of healthcare organizations.^3^ Rapid growth of aging population not only increase the demand for primary health care and long-term care, but also the need for well-trained personnel and age-friendly environments.

Kydd and Fleming described age discrimination as “arbitrary decisions concerning people because of their ages”.^4^ People in different age groups may experience various forms of discrimination. In this article, we specifically focus on discrimination because of old age.

The proportion of people aged 55 years and over who have experienced discrimination varies in different countries, e.g., 63.1% in Bulgaria, 60.3% in the Czech Republic, and 23.6% in Denmark.^5^ In a study that included 7500 men and women aged ≥ 52 years in England, about 33% of the participants claimed exposure to ageism.^6^ Dobrowolska et al studied ageism in healthcare institutions and reported that 30% of older adults in hospitals experienced age discrimination and 47% of students in medicine and nursing witnessed such discrimination by physicians.^7^ A study in Iran reported ageist attitude by 60.6% of nurses providing care to older adult patients.^8^

Ageism towards older people is prevalent and recognized as the main threat to active aging and a major public health concern.^9^ It may result in chronic stress and increase the risk of chronic diseases and mortality, as well as negatively affecting physical and psychosocial outcomes in older adults.^10^ Several studies have found that ageist attitude and stereotyping have negative effect on older adults in various domains, including memory and cognitive function,^11^ health,^12^ work performance,^13^ and even the will to live.^14^ Negative views on the aging act as self-fulfilling prophecies by encouraging people to classify and recognize age discrimination.^15^

The current rapid population aging is unprecedented in history and has introduced new issues, such as changing peoples’ perceptions and expectations of older people. This in turn results in negative attitudes and discrimination towards older people.^5^ In early 2021, the World Health Organization (WHO) released a report that triggered a global discussion on the incidence and effects of ageism in response to changes resulting from the COVID-19 pandemic.^16^ In that report, the WHO recognized ageism as a form of discrimination that is socially accepted. The WHO also underlined the importance of research studies and the unique image of each country since ageist attitude varies in different countries.^17^ It has been reported that societal behavior and expectations from older people are influenced by peoples’ perception of old age.^18^ Therefore, studying societal attitudes toward older people can help in the design of measures that prevent discriminatory practices.^17^ Furthermore, one of the important causes of age discrimination is a lack of awareness about what ageism is and what attitudes and behaviors may imply age discrimination, especially in clinical environments.^7^ Therefore, it is necessary to increase knowledge and awareness of those attitudes and behaviors that the older people perceive as age discrimination to reduce perpetration of age discrimination in clinical settings by utilizing some related studies. To date, most studies conducted on age discrimination have been based on a quantitative design.^6,19^ Although there are many quantitative empirical studies on ageism in the healthcare system, the majority have limitations due to the use of tools such as Kogan’s People Scale. This scale is designed to measure general attitudes toward older people, but it does not take into account the context of healthcare setting. Overall, there is very limited understanding of the expressions and manifestations of ageism.^20^ On the other hand, qualitative content analysis is a meaningful method to provide broad, in-depth insights about a phenomenon, its etiology, and its manifestations based on the perspective and experience of those involved.^20,21^ Many qualitative studies on the concept of age discrimination in clinical settings have focused on health professionals’ perspective,^8,22,23^ whereas age discrimination from the perspective of hospitalized older adults has received less attention. It is important to understand the concept of age discrimination in the context of specific cultural parameters and populations. This paper sets out to explore the concept of age discrimination perceived by hospitalized older adult patients.

## Material and Methods

### Participants and setting 

The present exploratory qualitative study was conducted from October 2019 to May 2020 at two teaching hospitals in Shiraz and Tehran, Iran. The target population was older adult patients hospitalized in various units of the hospitals, their family caregivers, and healthcare team (nurses and physicians). The participants were selected using maximum variation sampling according to key demographic characteristics such as age, education level, length of hospital stay, and type of hospital unit. The purposive sampling method was used to recruit participants and the sampling continued until data saturation, i.e., no new category could be extracted. In this regard, no new data were obtained beyond the 18^th^ interviews.

The inclusion criteria for the patients were aged ≥ 60 years, a minimum of 3 days of hospitalization, mental and physical ability to participate in an interview, willingness to take part in the study, and verbal ability to provide rich information about the subject of our study. The inclusion criteria for the family caregivers were aged > 18 years, being a family member of the patient, practical caregiving experience, accompanying the patient for a minimum of 3 days during the current hospitalization, the ability to provide rich information about the subject of our study, mental and physical ability to participate in an interview, and willingness to take part in the study. The inclusion criteria for the healthcare team were being knowledgeable about caring for older adult patients (at least 5 years of clinical experience) and willingness to take part in the study. Based on the inclusion criteria, a total of 21 individuals were recruited, namely older adult patients (n = 12), family caregivers (n = 5), nurses (n = 3), and a physician.

### Data collection

All participants were fully informed about the study objectives and methods, interview procedures, and the organization sponsoring the study. In addition, the confidentiality of any disclosed information was guaranteed and the procedure for possible withdrawal from the study was described. Written informed consent was obtained from all the participants.

Following approval from the person in charge of the hospital unit, the interviews were held in a private room of the hospital. The third author (a PhD candidate in Community Health Nursing with experience in conducting interviews) was assigned to interview the older adult patients. To fine-tune the interview guides, a pilot interview with a sample of each group was conducted by the third author prior to the main interviews. The modified version of the interview guides were then reviewed in a group meeting involving all authors (Supplementary file 1). The participants of the pilot interviews were not included in the actual study. The items of the interview guides covered topics such as participants’ perception, understanding, and description of age discrimination against hospitalized older adult patients as well as any past or current practical experience of ageism.

Each interview started with a set of general questions followed by probing questions for clarification and to extract additional information, e.g., ‘What is your understanding of age discrimination against hospitalized elderly patients? How would you define it?’ ‘Have you experienced ageism by healthcare team in the hospital?’ ‘During current or previous hospital stays, did you witness age discrimination by medical team against other elderly patients?’ The response of a participant to these questions tuned the questions for the next interview. To improve the clarity of information, follow-up questions were used as necessary.

Upon permission from the participants, the interviews were audio-reordered and transcribed verbatim at the most opportune time. In addition to the pilot interviews (n = 3), a total of 21 interviews were carried out. Each interview lasted between 40 to 70 minutes. Demographic characteristics of the participants are presented in Table 1.

**Table 1:** Demographic characteristics of the participants

**No.**	**Participation**	**Age (y)**	**Gender**	**Education**	**Ward**	**Length of hospital stay (days)**
P1	Patient	65	Male	Illiterate	Medical	6
P2	Patient	60	Female	Elementary school	Medical	7
P3	Patient	74	Female	Secondary school	Emergency	3
P4	Patient	68	Male	Illiterate	Surgery	7
P5	Patient	72	Male	Bachelor’s degree	Surgery	12
P6	Patient	80	Female	Illiterate	Medical	17
P7	Patient	76	Female	Secondary school	Medical	5
P8	Patient	64	Male	Elementary school	Medical	20
P9	Patient	72	Male	Illiterate	Surgery	5
P10	Patient	78	Female	Illiterate	Medical	8
P11	Patient	70	Female	Illiterate	Surgery	12
P12	Patient	62	Female	Secondary school	Medical	8
P13	Family member	36	Female	Master’s degree	CCU	7
P14	Family member	46	Female	Bachelor’s degree	Medical	8
P15	Family member	58	Male	Secondary school	Emergency	3
P16	Family member	47	Female	Diploma	Medical	15
P17	Family member	42	Female	Diploma	Emergency	16
P18	Nurse	34	Female	Bachelor’s degree	Medical	-
P19	Nurse	42	Female	Bachelor’s degree	Surgery	-
P20	Nursing director	36	Female	Master’s degree	Nursing Office	-
P21	Doctor	42	Male	GP	ICU	-

### Data analysis

Data were collected through 21 face-to-face, semi-structured, in-depth interviews and the data were analyzed using the qualitative content analysis method as described by Elo and Kyngäs.^24^ Initially, all interviews were transcribed verbatim and read for a thorough understanding and immersion in the data. In the second step, semantic units were organized and coded. The resulting primary codes were grouped based on similarities and sub-categories were deduced. Sub-categories were then assessed and grouped in terms of similarities to deduce generic categories. These were also assessed and grouped to define the main categories. Finally, the resultant categories were presented in a tabular format together with full descriptions. Note that the process of labeling the categories was flexible and altered after analyzing the data from each interview until data saturation.^24^ Data were organized using the MAXQDA 2007 software.

### Rigor

The four criteria proposed by Lincoln and Guba^25^ were used to demonstrate the trustworthiness of our study. The credibility and confirmability dimensions were fulfilled through prolonged engagement (7 months) with the subject of the study to ensure in-depth understanding of the concept. Moreover, to improve the credibility criterion, the initial coding of data (by the third author) was independently reviewed by each member of the research team followed by a review by all research members. In addition, coded data were assessed by two qualitative researchers (PhD nursing faculty members) blinded to the study process. Data triangulation was also used, including persons (semi-structured interviews with a variety of individuals; older adult patients, family caregivers, nurses, and physicians), time (conducting interviews during different hospital shifts), and space (various units of the two hospitals). Finally, the results were cross-checked by the participants for accuracy, completeness, and conformity of the interpretations with their experiences as well as ensuring correct coding. Accordingly, the feedback from the participants was used to modify some items. To ensure confirmability and dependability of data, specific actions were taken to use the correct interview technique, prepare accurate field notes and documentation, and use a peer-review process. To fulfill the transferability dimension, we clearly described demographic information, research environment, interview technique, and data collection and analysis process.

## Results

Twenty-one individuals from two hospitals were recruited for face-to-face, semi-structured, in-depth interviews. The participants included patients (n = 12, aged 60-80 years), family caregivers (n = 5, aged 35-58 years), nurses (n = 3, aged 34-42 years), and a physician (n = 1, aged 42 years). Hospitalization was typically due to heart and lung diseases, diabetes, or gastrointestinal disorders with a hospital stay varying from 3 to 20 days. Demographic characteristics of the participants are presented in Table 1.

Twenty-one interviews were conducted to assess the participants’ perceptions of ageism in healthcare settings. Data analysis resulted in 4 main categories, 8 generic categories, and 21 subcategories (Table 2). The main categories were injustice perceptions, interactional injustice, procedural injustice, and organizational injustice.

**Table 2:** Categories and sub-categories extracted from qualitative data analysis about the perception of older adult patients on age discrimination

**Main category**	**Generic category**	**Sub-category**
Injustice perceptions	Professional ageism	Misconception about aging
		Age-biased attitudes
	Disability discrimination	Rejection
		Low priority patients
Interactional injustice	Epistemic injustice	Informational prejudice
		Marginalization
	Discriminatory communication style	Aggression and hostility
		Superficial communication
		Non-individualized communication
Procedural injustice	Unfair care	Unmet physical care needs
		Delayed health care
		Uniformity of care
		Social discrimination
	Medical malpractice	Over investigation
		Misdiagnosis and incorrect treatment
Organizational injustice	Unsupportive hospital environment	Improper physical structure
		Insufficient geriatric facilities and medical equipment
	Lack of age specific health care policy	Shortage of geriatric professionals
		Lack of age-related admission policies
		Lack of geriatric screening procedure
		Lack of geriatric clinical practice guidelines

### Injustice perceptions

Two generic categories were identified for injustice perceptions, namely professional ageism and disability discrimination. Elderly patients perceived the healthcare team as having an ageist attitude and misconception about older people. They believed that the personnel tend to pay more attention to patients with better physical and mental health and gave low priority to the needs of older adult patients.

#### Professional ageism

Sub-categories associated with professional ageism were misconceptions about aging and age-biased attitudes.

*Misconceptions about aging:* Patients and their family caregivers believed that misconceptions about old age among healthcare professionals resulted in negligence and discrimination in the provision of care and treatment for older adult patients. Older people are perceived as disobedient, pretensive, attention-seeking, stubborn, uncooperative, dependent, and forgetful. A participant stated:

“*Despite excruciating pain, nurses presumed that my old mother was faking the pain. They did not believe her even when she was in tears due to pain and considered it as a way of seeking attention. It is incomprehensible that they do not trust it when older adult patients say they are in pain.*” [36-year-old family caregiver]

The above statement was in line with the practical experience of the physician and nurses. A participant stated:

“*Some members of our healthcare team have a negative attitude toward older adult patients. They treat them as if they are children, indifferent to their personal health, and attention seekers.*” [42-year-old physician]

Older adult patients and their family caregivers perceived that nurses drew a parallel between old age and pain, disability, and inevitable death. As a direct result, they felt that caring for older adult patients is futile, not important, and time-consuming. A participant stated:

“*The healthcare team does not seem to care for older adult patients at all. They probably think these individuals are too old and unworthy of treatment time and costs since they will soon die of old age.*”[47-year-old family caregiver]

*Age-biased attitudes:* A typical discriminatory attitude by healthcare team is that they perceive all old people to suffer from poor eyesight, hearing, and memory as well as having learning and functional disabilities. A participant stated:

“*The healthcare team treats us as if we are dumb, forgetful, ineducable, and suffer from memory loss. They shout at us as if we are deaf. In the presence of my daughter, they discuss things with her instead of me.*”[64-year-old male patient]

Another misperception by healthcare team is to associate the patient’s age with issues such as pain, loss of appetite, and various physical and mental problems. Some participants felt that such misperceptions prevented timely diagnosis and treatment of older adult patients. A participant stated:

“*For a while, our relative did not feel well and complained of body aches,*loss of appetite*, and drowsiness. We approached various physicians and they all associated these symptoms with old age. They prescribed over-the-counter painkillers which did not have any effect at all, and again they put it down to old age. When her condition worsened, we had to take her to the hospital. Regretfully, after multiple tests, she was diagnosed with cancer.*”[42-year-old family caregiver]

#### Disability discrimination

This generic category was related to situations where older adult patients with serious physical illness were less likely to receive appropriate health care. The associated sub-categories were rejection and low-priority patients.

*Rejection:* Some participants indicated that the healthcare team preferred to invest their time to care for patients with better physical and mental abilities rather than frail older people. A participant stated:

“*I did observe situations where nurses refused to accept care responsibility for older adult patients during assignments because of personal preference or the patient’s age and health condition.*”[47-year-old family caregiver]

A nurse confirmed the above statement and testified:

“*Older adult patients are generally uncooperative and tend not to accept medications or refuse to eat. This is frustrating for us and consequently, we prefer not to accept such patients because it is too time-consuming.*”[34-year-old nurse]

*Low-priority patients:* Some participants indicated that older people are considered a low priority for diagnostic procedures and treatment compared to younger patients. This is particularly the case when the healthcare center is short of equipment. A participant stated:

“*I am very much concerned that my old mother will not receive proper treatment in the hospital and will be categorized as a low-priority patient.*”[42-year-old family caregiver]

### Interactional injustice

As clearly stated by all participants, older people experience interactional injustice more profoundly than other patients. Generic categories associated with this main category were epistemic inequality/injustice and discriminatory communication style. The participants of our study felt that the healthcare team avoided proper interaction with older adult patients, withheld essential information, and ignored them during clinical rounds. They also believed that the healthcare team had an aggressive and hostile attitude toward older adult patients and only established short, superficial, and impersonal communication with these patients.

#### Epistemic inequality/injustice

Epistemological inequality is defined as unfair distribution of information and excluding patients from the decision-making process and healthcare counseling.

*Informational prejudice:* Unfair distribution of information is mainly associated with the lack of patient-physician communication during clinical examinations. This could be in the form of not talking to older adult patients or not providing them with the necessary information. Two participants stated:

“*The healthcare team approached my daughter to obtain information about my medical condition. They asked her about my illness, duration, the extent of pain, and my appetite. They did not listen to my response. It seems they do not value the feedback from older people.*”[72-year-old patient]

“*The healthcare team does not give any information about the recovery process of our relatives or the length of hospital stay. If we ask nurses to summarize the contents of our grandfather’s dossier, they claim to be busy and refer us to his physician, but we never get the opportunity to see the physician.*”[42-year-old family caregiver]

Another shortcoming stated by some participants was related to inadequate information about post-discharge care of older adult patients. All that was given were patient-relevant educational pamphlets.

*Marginalization:* Some participants indicated that older adult patients are incorrectly marginalized by the healthcare team. For instance, their opinion on the treatment process is not taken into account and the presence of a family caregiver during doctor’s rounds is prohibited. Two participants stated:

“*The healthcare team does not take us seriously. They address their questions to my children rather than directly to me. I do not understand why I am not allowed to get involved and learn more about my illness. The right to know has nothing to do with age.*”[60-year-old patient]

“*Doctors conduct their rounds with the residents and nurses during which information exchange only takes place between them and patients are ignored. It is as if we are guinea pigs in an educational session.*”[78-year-old patient]

#### Discriminatory communication style

Age-based discriminatory communication was clearly stated during the interviews with older adult patients. The associated sub-categories were aggression and hostility, superficial communication, and non-individualized communication.

*Aggression and hostility:* Some participants experienced aggressive and hostile behavior by the healthcare team, particularly by nurses. Two participants stated:

“*Some nurses always seem to be bad-tempered and hasty. They do not show any respect for older people nor appropriately answer their questions. They are often rude and impatient.*”[65-year-old patient]

“*Such patients are generally slow when doing things or comprehend instructions. Instead of shouting at them or forcing them to do things quickly, nurses should show understanding and compassion.*”[64-year-old patient]

*Superficial communication:* This sub-category is related to the communication style of the healthcare team toward older adult patients. According to some participants, discriminatory communication was illustrated by superficial communication or the complete avoidance of any conversation. A participant stated:

“*The healthcare team shows no respect for older adult patients. If they ever talk to us, they do it in a short and sharp manner or while walking out of the room.*”[72-year-old patient]

*Non-individualized communication:* Lack of attention in choosing a communication style appropriate to the specifics of each older adult patient was also mentioned by some participants. A participant stated:

“*The healthcare team does not seem to appreciate the difference between young and senior patients and approach both in the same manner. A young patient may tolerate a harsh tone of voice, but older people are more sensitive and require a different style of communication. I think the healthcare team needs to follow a communication training course.*”[72-year-old patient]

### Procedural injustice

Generic categories associated with procedural injustice were unfair care and medical malpractice. The patients in our study believed that the healthcare team addressed their needs either with a delay or simply ignored them altogether. The personnel seemed to treat older adults in the same manner as any other patients without any consideration for their specific needs. Apparently, the socio-economic status of patients influenced the level of care provided by the healthcare team. In addition, they often performed unnecessary therapeutic-diagnostic procedures for which the results were at times incorrect.

#### Unfair care

This generic category included items such as delayed health care for the older compared to young patients, unmet physical care needs, uniformity of care for all patients irrespective of their age and specific needs, and discrimination based on the social and financial status of older adult patients.

*Delayed health care:* Some participants associated unfair care with delays in responding to the medical and nursing care needs of older adult patients. A participant stated:

“*To examine my foot, the physician removed my foot bandage in the early evening with the promise that someone would come soon to put a new bandage on. To my surprise, over 8 hours passed and nobody attended to my wound. I repeatedly asked nurses and reminded them of the risk of infection, but none paid any attention.*”[65-year-old patient]

Some participants also mentioned delays in addressing patients’ care needs, especially personal care and health needs. A participant stated:

“*An older adult patient repeatedly sat up in the hospital bed and requested assistance to go to the toilet, but the nurse was busy and each time he was told to lay flat and wait for a bedpan. In the end, the nurse did not bother to respond and the poor patient wetted himself. They even did not change his clothes and he slept in a dirty bed throughout the night until his family caregiver arrived in the morning.*”[36-year-old family caregiver]

*Unmet physical care needs:* Some participants described unfair care as discrimination in fulfilling the basic and physical needs of older adult patients compared to other age groups with similar problems. Two participants stated:

“*During her ICU stay, my mother was fed an inadequate diet at irregular times. Nurses did not check if she was hungry and were often impatient with her slow eating habit.*”[36-year-old family caregiver]

“*Nurses take advantage of family caregivers by passing on their responsibility to them. It needs two people to change the clothes of a heavy patient, like my mother. I had to do it on my own since the nurses did not help at all. They would not even allow another family member to enter for assistance.*”[46-year-old family caregiver]

*Uniformity of care:* Another aspect of unfair care was related to the uniformity of care for all patients irrespective of their age and needs. A participant stated:

“*Nurses do not fully appreciate that the age of a patient is a critical factor. The older the patient, the higher the care needs. Older adult patients are very sensitive, weak, frail, and need more attention and care compared to younger patients.*”[58-year-old family caregiver]

*Social discrimination:* Social and economic status of a patient (e.g., social class and descendant) contributed to discrimination in healthcare services. It appeared that patients with a higher level of education or wealth were treated better and with more respect. A participant stated:

“*There are many patients from rural areas, but none receive the same level of respect and preferential treatment as wealthy individuals. Favoritism also plays a role, without which common patients stand no chance of receiving proper care.*”[80-year-old patient]

#### Medical malpractice

This generic category included items such as “Misdiagnosis and incorrect treatment” and ‘Over-investigation”

*Misdiagnosis and incorrect treatment:* Some participants claimed medical negligence through misdiagnosis and subsequent incorrect treatment leading to unfavorable outcomes. A participant stated:

“*After a painful foot infection, I was unnecessarily hospitalized and the medical team conducted many tests. I kept pointing their attention to my foot infection, but they kept on performing other tests. I went through a painful process and in the end, they found no major issues other than my foot infection. But by that time, it was too late, and the infection had reached the bone requiring foot amputation.*”[72-year-old patient]

*Over-investigation*: Some participants indicated that the medical team performed immethodical and unnecessary invasive/non-invasive diagnostic and therapeutic procedures many times (e.g., colonoscopy, endoscopy). A participant stated:

“*They do not perform a sonography first-time-right. One must do a test multiple times and repeatedly go through the whole process of test registration and queuing. This is not only challenging for older adult patients, but also time-consuming for both test center and physician*.” [60-year-old patient]

### Organizational injustice

This main category was associated with age discrimination due to decisions made by health policymakers and planners as well as hospital managers. The associated generic categories were unsupportive hospital environment and the lack of age-specific healthcare policy. The participants in our study stated that the physical structure of the hospital environment was not appropriate for older adult patients and the provided medical equipment and facilities were inadequate. They also mentioned some key organizational issues related to such patients, such as shortage of specially trained personnel, lack of screening procedures and clinical practice guidelines, and the absence of age-related policies during the admission process.

#### Unsupportive hospital environment

The associated sub-categories of unsupportive hospital environment were improper physical structure and insufficient geriatric facilities and equipment.

*Improper physical structure:* Some participants indicated that the physical environment of hospitals (i.e., architectural design, structure, space) is not suitable for older adult patients and may cause injuries. A participant stated:

“*The hospital is cramped, the floors are slippery, and there are not enough toilets and bathrooms. The lighting system is poor and the wall guards are not designed for us and are inadequate to keep our balance. Mattresses are old and not suitable for older people who might develop bedsores after days or weeks of hospitalization. Both young and old patients are crammed into one room while having different needs. Hospital managers should address these issues.*”[64-year-old patient]

A patient commented on the restricted physical space in the hospitals and its effect on the morale of older adult patients and stated:

*“The hospital is too crowded and annoying. Rooms are too small and the beds are too close to each other. It is chaotic having patients, caregivers, and nurses all running about in the corridors. All these negatively affect older adult patients who need a calm environment.*”[74-year-old patient]

*Insufficient geriatric facilities and medical equipment:* Some participants commented on the lack of sufficient geriatric facilities in hospitals and specifically mentioned alternating pressure mattresses for immobile patients. A participant stated:

“*Since my relative could not walk and was prone to develop bedsores, I requested an alternating pressure mattress. But the response was that such mattresses are only for severe cases and we do not have enough of them.*”[47-year-old female caregiver]

Additional examples of insufficient geriatric facilities were the limited number of wheelchairs or their deplorable condition, lack of electric adjustable beds, malfunctioning beds, the use of stretchers in the emergency unit due to lack of beds, and the keeping of older people in a wheelchair for hours instead of laying them in bed. A participant stated:

“*Our patient had to be discharged without being treated because of malfunctioning equipment.*”[47-year-old family caregiver]

#### Lack of age-specific healthcare policy

An aspect of organizational injustice was the lack of specific healthcare policies for older adult patients. Such a shortcoming resulted in the shortage of highly trained and specialized individuals dedicated to caring for older adult patients, the absence of admission and hospitalization guidelines, the lack of procedures to screen older people for common illnesses, and the lack of clinical guidelines.

*Shortage of geriatric professionals:* Some participants indicated a shortage of skilled and specialized personnel dedicated to caring for older adult patients. A participant stated:

“*Some nurses do not even know how to locate a vein to draw blood. We had to wait until an experienced nurse performed the venipuncture. I believe a geriatric team should be responsible for the care of older adult patients.*”[36-year-old family caregiver]

Two nurse participants commented on this issue and stated:

“*Most of us are not professionally trained to care for older adult patients and our behavior toward such patients could be misinterpreted as disrespectful. We do not claim to have the same level of expertise as a geriatric nurse*.” [42-year-old nurse]

*Lack of age-related admission policies:* According to some participants, another aspect of organizational injustice was the lack of specific guidelines for the admission and hospitalization of older adult patients. A participant stated:

“*There should be a specific procedure and priority hospitalization for the older people since they are physically and mentally more vulnerable compared to other types of patients. Because there were no free beds, our relative was not attended to but placed on a stretcher in the corridor for a few hours. He was then transferred to the emergency unit and it took them 3 days before transferring him to the appropriate ward. As a result, he developed bedsores which would probably not have occurred in a young patient.*”[36-year-old family caregiver]

*Lack of geriatric screening procedure:* Some participants indicated that the healthcare team does not pay attention to underlying problems, disabilities, and physical limitations (poor sight, hearing, memory, mobility) of older adult patients. A participant stated:

“*Older adult patients are very vulnerable and suffer from various physical limitations. We must provide the highest degree of care to prevent potential complications.*”[34-year-old nurse]

*Lack of geriatric clinical practice guidelines:* Nurses and physicians also acknowledged the need for hospital managers to formulate dedicated guidelines and procedures for the treatment and care of older adult patients. A participant stated:

“*From the physiological point of view, there is a distinct functional difference between older adults and young patients in terms of renal and digestive system function and response to medication. Nonetheless, all patients are treated in the same manner due to either the lack of knowledge or specific guidelines and procedures. This is a serious problem that we currently have to deal with*”[38-year-old nursing supervisor in charge of training]

## Discussion

In the present study, the perception of hospitalized older adult patients about ageism was assessed at two teaching hospitals in Shiraz and Tehran (Iran). Analysis of the interview data on age discrimination resulted in four main categories, namely injustice perceptions, interactional injustice, procedural injustice, and organizational injustice.

### Injustice perceptions 

Generic categories associated with injustice perceptions were professional ageism and disability discrimination. Professional ageism was characterized by negative attitudes and stereotypes toward age and generalization of age-related physical and mental limitations to all older people.

Some participants indicated that the healthcare team draws a parallel between old age and disabilities, and assumes all older people suffer from poor sight, hearing, and memory loss. Worst of all, the team views confusion, forgetfulness, and inability to learn and understand inherent to old age. These led them to treat older adult patients differently and consider caring for them pointless and time-consuming. Our findings were in line with other studies that reported misperceptions by healthcare team about old age and the inherent nature of certain symptoms with old age,^26^ treating an older adult patient is pointless,^27^ and they are not worthy of care.^28^ In another study, Heydari and colleagues stated that there are misconceptions about older people and that they are perceived incapable of adapting to new situations, ineducable, impatient, irritable, and bad-tempered.^29^ In a qualitative study, nurses’ attitudes and perception of older adult patients were reported to be negative, prejudiced, and biased.^29^ Age-biased attitude, i.e., generalization of age-related limitations (poor sight, hearing, and memory) to all old people, has also been reported in other studies.^30-32^ Such misconceptions can negatively affect healthcare decision-making and lead to incorrect diagnosis and treatment, neglect of older adult patients, and inequality in the provision of medical and nursing care.^31^ Therefore, it is important to create awareness to change the negative age stereotyping toward older adult patients to ultimately improve care for this age group.

According to our participants, the healthcare team tends to ignore frail older adults and categorized them as low-priority patients for diagnostic procedures and treatment. A previous study reported that discriminatory attitudes based on age and severity of disability can negatively affect the quality and quantity of older adults’ care as well as treatment outcome, leading to inequality in the provision of medical and nursing care.^31^

Provision of quality care to all patients irrespective of race, gender, age, and socio-economic status is one of the ethical responsibilities of healthcare professionals, particularly nurses as frontline healthcare workers.^33^ Ageist attitudes at any level of the healthcare system negatively affects the quality of care and ultimately the health outcome of older adults. It is therefore important to develop a culture of respect for older adults through continuing education programs. In this process, the use of social media and the creation of role models are strongly recommended. Also, considering the specific needs of older adult patients, healthcare policymakers and hospital managers should better balance the needs of elderly patients with care services, e.g., efficient allocation of nurses and hospital rooms to older adults. This could potentially lead to a reduced workload on healthcare personnel and may indirectly prevent age discrimination at workplace.

### Interactional injustice

The findings of the present study indicated that interactional injustice was in the form of epistemological inequality and discrimination both in caregiver/patient relationship and in communication. Epistemological inequality was identified as unfair discrimination in the provision of information to patients, exclusion from the decision-making process and from medical counseling.

Our participants stated that the healthcare team assumed that cognitive processing and working memory in older adults are too deficient to absorb given information; it is therefore pointless and a waste of time to share information with such patients. These findings were in line with a previous study reporting that physicians view the information provided by patients are often irrelevant and useless. Physicians perceive unreliable cognitive behavior and emotional instability of their patients are to the extent that their feedback and interpretations can be invalid.^34^ In a qualitative study among older adult patients, Naderi and colleagues reported similar findings.^35^ Another qualitative study reported that healthcare specialists tend to exclude older adult patients from discussions about their medical condition, instead they decide on their own or involve a younger family member.^20^ This is despite the fact that the involvement of older adult patients in the decision-making process would positively affect their satisfaction level and lead to better treatment outcomes.^36^ Since the lack of information is one of the main sources of fear and anxiety in patients, it is important that they are made aware of all necessary and practical information (diagnostic, treatment, and care) in an understandable language to reassure the patients.

Another aspect of interactional injustice was discriminatory communication with older adult patients and their family caregivers. Some participants experienced aggressive and hostile behavior by the healthcare team, superficial communication, and short task-oriented conversations. In a previous study, Huang et al also reported inappropriate communication styles between the healthcare team and older adult patients.^37^ Another study stated that older adult patients expect the healthcare team should primarily act based on the principles of good communication, take the concerns of older people into account, and share information and decisions with them.^38^

One of the basic and essential professional skills expected from healthcare teams, especially nurses, is effective interaction with all patients irrespective of age. Older people may be increasingly reliant on caregivers (friends or family) and the more people involved, the greater the risk of misunderstanding.^38^ Lack of knowledge and communications skills to appropriately interact with older adults as well as misconceptions about aging will negatively affect the performance of healthcare professionals. These results in older adult patients distrusting the healthcare team and behaving in a manner that resembles confusion, stress, and temperamental. Nonetheless, healthcare professionals incorrectly relate such behaviors to old age rather than their own shortcomings, which in turn exacerbates their ageist attitude.

### Procedural injustice

Procedural injustice or inequality in treatment and care procedures was another main category with two generic categories, namely unfair care and medical malpractice. Unfair care was associated with delays in responding to the care needs of older adult patients, e.g., personal care and health needs as well as delays in diagnostic-therapeutic and medical procedures, admission process, and transfer to appropriate wards. Other studies also reported inequalities in diagnostic-therapeutic procedures for older adult patients in various settings.^31,39^ In a systematic review, Ouellet and colleagues reported that older adults with acute myocardial infarction experienced more delays during pre-hospitalization compared to younger patients.^40^ Another review study found that the care needs of older adult patients, especially basic human needs, are often overlooked and neglected in care plans.^41^ Neglecting physical needs related to the daily life activities of patients is a common event in many health centers, to the extent that it has become a global nursing issue.^41,42^ In this regard, the WHO indicated that these omissions could directly or indirectly harm older adult patients and negatively affect healthy aging at home and in the community.^2^ Fulfilling the care needs of older adult patients is an ethical principle and undignified care is indicative of unmet care needs,^41^ which in turn could undermine the safety of these patients and negatively affect the treatment of their acute or chronic problems.

Misdiagnosis and over-investigation were also mentioned by our participants. Other studies reported similar results and showed that the rate of misdiagnosis was higher than any other medical errors.^32,33^ Misdiagnosis may lead to repeated clinical and paraclinical evaluations, which would not only endanger the health of older adult patients but impose excessive costs to the healthcare system.^43^

Given the patients’ rights charter and the rapid growth of the elderly population, it is essential to adequately address the therapeutic care needs of older adult patients. Otherwise, the condition of these patients already suffering from multiple diseases becomes even more complicated and imposes additional costs, time, and effort on the healthcare system.

### Organizational injustice

Organizational injustice was another aspect of ageism with two generic categories, namely unsupportive hospital environment and insufficient geriatric facilities and medical equipment. In line with our results, Ahmadi Teymourlouy and colleagues reported that some Iranian hospitals are not age-friendly and there are some deficiencies in the provision of care, preventive interventions, and overall health care of older adult patients.^44^ They also highlighted shortcomings in the interaction between the healthcare team and older adult patients, resource allocation policies, shortage of specialized personnel for geriatric care, and physical-medical facilities for older adult patients. A study by Heidari and Mardani-Hamooleh also reported a shortage of specialized personnel for geriatric care in the Iranian healthcare system.^45^ Various studies have also reported issues related to a long-term stay of older adult patients at the emergency unit and subsequent transfer to appropriate wards. This was particularly the case for patients with serious clinical conditions and chronic illnesses. With a specific focus on older adult patients, the WHO emphasizes establishing age-friendly hospitals that provide a dedicated outpatient department, admission process, and priority system. It also recommends healthcare providers to adhere to operational guidelines for the care needs of older adult patients.^46^

Considering that most people admitted to healthcare centers are older adults with specific conditions, it is essential to pay attention to their needs at the organizational level. On the other hand, possible inequalities due to inadequacies in different organizations (physical environment, facilities and resources, policies, and guidelines) may directly or indirectly impact the perception of ageism and the performance of healthcare teams. This underlines the need for specific policies and organizational infrastructure dedicated to older adult patients.

The participants were recruited among older adult patients hospitalized in only two teaching hospitals. It is recommended that further studies on this topic include a wider range of health centers. It is also recommended that future research should include a match between patients and healthcare professionals to solicit a better understanding of ageism in healthcare settings.

## Conclusion

The results of the present study indicated that older adult patients and their family caregivers consider age discrimination in hospitals as the cause of unfair care. In addition, they viewed ageism, interactional injustice, and young-oriented health policies as the main cause of violation of older adult patients’ rights.

Our findings highlight the need for the transformation of hospitals and other health centers toward an age-friendly environment. This process primarily requires educating the healthcare team to change their misconceptions about older adult patients and to learn how to interact with them. In addition, the healthcare team should be aware of the natural process of aging, learn about active and healthy aging, management of geriatric disorders, pharmacokinetics and pharmacodynamics of drugs related to the advanced stage of life, physiological changes that occur with aging, the health risks and specific care needs of older adult patients, and bias against socio-economic status. Such awareness, attitude, and practice would ultimately improve the effectiveness, efficiency, and appropriateness of healthcare services for older adult patients. As a prerequisite, it is essential to move from a disease- and organ-based model to a biological-psychological-social model and comprehensive interdisciplinary care.

## Acknowledgments

We would like to thank the participants for their time, effort, and contribution to the study.

## Authors’ contributions

SG and MS contributed to the study conception/design, data analysis and thorough interpretation, drafting, and critical review of the article. ZN and FH contributed to data acquisition/analysis. All authors have read and approved the final version of the manuscript.

## Funding

The current study was financially supported by the Research Vice-Chancellor of Shiraz University of Medical Sciences, Shiraz, Iran (grant number: 1398-01-86-20289). The funder had no role in the design of the study and collection, analysis, and interpretation of data and in writing the manuscript.

## Ethical approval

Ethical approval was obtained from the local Ethics Committee of Shiraz University of Medical Sciences (IR.SUMS.REC.1398.889). Eligible participants were informed about the study objective and the voluntary nature of their participation. Written informed consent form was filled out by all the participants.

## Competing interests

The authors declare that there is no conflict of interest.

## Supplementary Materials

Supplementary file 1. Semi-structured interview guide.
