# Supplementary material for: Age discrimination perceived by hospitalized older adult patients in Iran: A qualitative study
Source: Health Promot Perspect. 2022 May 29;12(1):45–55. doi: 10.34172/hpp.2022.07 (PMC9277281; doi:10.34172/hpp.2022.07)
Supplement: Supplementary file 1 — Semi-structured interview guide. [file hpp-12-45-s001.pdf]

Supplementary file 1. Semi-structured interview guide

<table>
<tr><th colspan="2">Interview guide 1: Older adult patients</th></tr>
<tr><th>General question</th><th>Probing questions</th></tr>
<tr><td>What is your understanding of age discrimination against hospitalized older adult patients? How would you define it?</td><td rowspan="2">Please provide an example of what you perceive as an ageist attitude.</td></tr>
<tr><td>What is the first thing that comes to your mind when one mentions ageism?</td></tr>
<tr><td>Have you experienced ageism by healthcare teams in the hospital?</td><td>What did exactly happen? How did you feel and react? At which point did you sense being subjected to ageism? Do you think it was meant to harm you?</td></tr>
<tr><td>Have you in the past experienced age discrimination by any member of the healthcare team in the hospital?</td><td>Please explain in detail. What happened between you and the healthcare staff? When, where, and how did it happen? What was your response? How did you feel then?</td></tr>
<tr><td>During current or previous hospital stays, did you witness age discrimination by medical teams against other older adult patients?</td><td>Please describe the situation in detail.</td></tr>
<tr><td>Are there other instances of ageism against hospitalized older adult patients that you would like to mention?</td><td>Please expand.</td></tr>
<tr><th colspan="2">Interview guide 2: Family caregivers</th></tr>
<tr><th>General questions</th><th>Probing questions</th></tr>
<tr><td>What is your understanding of age discrimination against hospitalized older adult patients? How would you define it?</td><td rowspan="2">Please provide an example of what you perceive as an ageist attitude.</td></tr>
<tr><td>What is the first thing that comes to your mind when one mentions ageism?</td></tr>
<tr><td>Do you recall ageism or age-based inequality by healthcare teams against your older adult patient during his/her current hospital stay?</td><td>What did exactly occur? What did happen between your older adult patient and the healthcare team? At which point did you sense that your older adult patient has been subjected to ageism? Do you think they did it with the intention of harming your older adult patient?</td></tr>
<tr><td>During the previous hospital stay of your older adult patient, did you witness age discrimination by healthcare teams against him/her?</td><td>Please describe the situation in detail.</td></tr>
</table>

During your patient's previous or current hospital stay, did you witness age discrimination by healthcare teams against other older adult patients?	Please describe the situation in detail.
Are there other instances of ageism against hospitalized older adult patients that you would like to mention?	Please expand.
**Interview guide 3: Healthcare team**	
What is your understanding of age discrimination against hospitalized older adult patients? How would you define it?	Please provide an example of what you perceive as an ageist attitude.
What is the first thing that comes to your mind when one mentions ageism?	
While providing care to older adult patients, do you recall having consciously or unconsciously discriminated against them simply because of their age?	Please describe the situation in detail. What did happen between you and the older adult patient? Do you think you did it with the intention of harming the older adult patient?
Have you ever witnessed age-based discrimination against older adult patients by your colleagues while providing care?	Please described in detail.
Are there other instances of ageism against hospitalized older adult patients that you would like to mention?	Please expand.
